# Rheology and Tribology of Ethylcellulose-Based Oleogels and W/O Emulsions as Fat Substitutes: Role of Glycerol Monostearate

**DOI:** 10.3390/foods11152364

**Published:** 2022-08-07

**Authors:** Ruoning Zhang, Yanhui Zhang, Jingjing Yu, Yanxiang Gao, Like Mao

**Affiliations:** Key Laboratory of Functional Dairy, Ministry of Education, College of Food Science & Nutritional Engineering, China Agricultural University, Beijing 100083, China

**Keywords:** glycerol monostearate, ethylcellulose, oleogels, emulsions, rheology, tribology

## Abstract

Rheological and tribological properties of oleogels and water-in-oil (W/O) emulsions are important for application in fat substitutes. This study investigated the roles of glycerol monostearate (GMS) in tailoring the structural, rheological and tribological properties of ethylcellulose (EC)-based oleogels and W/O emulsions as potential fat substitutes. The addition of GMS contributed to more round and compact oil pores in oleogel networks. The oleogel with 5% GMS had higher crystallinity, leading to solid state (lower tanδ value), mechanical reversibility (higher thixotropic recovery), but a brittle (lower critical strain) structure in the samples. GMS gave the oleogels and emulsions higher oil binding capacity, storage modulus and yield stress. Under oral processing conditions, GMS addition contributed to higher textural attributes and viscosity. Friction coefficients in mixed and boundary regions of oleogels and emulsions were reduced with the increase in GMS content from 0~2%, but increased with 5% GMS. Rheological and tribological properties of lard, mayonnaise and cream cheese can be mimicked by EC oleogels with 5% GMS, or emulsions with 2% GMS and 2–5% GMS, respectively. The study showed the potentials of oleogel and W/O emulsions in designing low-fat products by tuning the structures for healthier and better sensory attributes.

## 1. Introduction

Excessive consumption of saturated fats, trans-fats and total fats is linked to a higher risk of chronic metabolic diseases. Much research has been carried out to design fat substitutes, e.g., using protein and polysaccharide to substitute for fats. Nevertheless, fats contribute to the sensory properties of foods, and a complete exclusion of fat would result in products with unfavorable texture and flavor attributes [1]. Oleogelation, which transfers oil from liquid state to semi-solid state without changing its composition, shows potential as fat substitute [1,2]. Oleogels were not only proved to imitate the fat functionality of saturated fat without trans-fatty acids, but were also effective in inhibiting the association of water droplets, and improving resistance to environmental stresses [3,4]. However, the oil content of oleogels were still high. Considering this, the incorporation of water in oil to form W/O emulsions is a promising approach to reduce fat [1,5,6]. Compared to O/W emulsions, W/O emulsions showed favorable organoleptic properties compared to full fat, with oil as the external phase [7]. Ethylcellulose (EC) is the only known food grade polymer-type oleogelator, which originates from natural resources and has good gelling capacity. However, the oleogels stabilized solely by EC were inhomogeneous and had poor oil holding capacity [8]. Glycerol monostearate (GMS) is the most widely used food grade emulsifier, and also has gelling capacity in oil. Studies reported that GMS incorporation resulted in plasticization of EC oleogels [9]. Although EC based oleogels were widely investigated, water-in-oleogel emulsions were less reported as potential fat substitutes.

Rheology and tribology have been employed to clarify the interactions between the structural elements during multiscale deformation. On the other hand, rheo-tribological information can be used to simulate oral processing behaviors of fat [10]. Typical W/O emulsions exhibited shear-thinning behavior and predominantly elastic characteristics, and the surfactant type and content had large effects on the rheological properties [11]. Ushikubo et al. (2014) investigated the W/O emulsions formulated with three emulsifiers (polyglycerol poly-ricinoleate (PGPR), Span 80 and lecithin), and found the viscosity of emulsions was dependent on the chemical affinity of the hydrophobic moieties of the emulsifier and the oil [12]. Generally, W/O emulsions had low elasticity and high mobility due to the low solid content, and oleogelation can greatly affect the rheological properties. Oleogelation could allow stiffer, stronger, and more brittle properties of the emulsions, presenting similar appearance and spreadability as mayonnaise [13,14]. On the other hand, the interface might have different interactions with the oleogel networks, leading to various filling effects of the water droplets. Some oleogelators can adsorb onto the interface to enhance interface thickness, and thus increase the storage modulus [6]. In the wax-based W/O emulsions, monoolein promoted the adsorption and crystallization of wax at the interface, and the emulsions presented higher storage modulus with increase in water content [15].

Oral processing undergoes a transformation from rheology-dominated behaviors to tribology-dominated behaviors (e.g., lubrication) in the later stages. The apparent viscosity and tribology were paramount in the description of sensory properties (creaminess, smoothness, etc.) [16]. In O/W systems, tribology was affected by the droplet size, droplet rigidity and oil content. Typically, the friction coefficients of emulsions decreased with increased fat concentration at low entrainment speed [17], and emulsions had much lower friction coefficients than the individual oil phases [18]. Our previous study showed that oleogelation contributed to higher friction coefficients of emulsions in mixed and hydrodynamic lubrication regions in O/W emulsions [10]. However, information about the tribological properties of oleogels and W/O oleogel emulsions is quite limited. Studies showed that the W/O emulsion with 30% water presented hydrodynamic film of similar thickness to that of pure oil [19]. Moreover, the volume of dispersed phase can affect tribological property via the change in the dynamic viscosity [7]. Unlike O/W systems, the viscosity ratio of oil phase to aqueous phase in W/O emulsions had no effect on the tribology in the hydrodynamic regime, while this ratio significantly affected boundary and mixed regimes [7].

In this study, oleogels and W/O emulsions were prepared based on ethylcellulose (EC), and the rheological and tribological properties were adjusted by glycerol monostearate (GMS). Rheological and tribological properties of the samples were also compared with those products containing saturated fats (lard, mayonnaise and cream cheese). The study aimed to understand the relationship between the structures and oral perception behaviors of W/O emulsions, for the better development of fat-reduced food.

## 2. Materials and Methods

### 2.1. Materials

Ethylcellulose (EC) (9–11 mPa·s, 5% in toluene/ethanol, 48% ethoxyl) was purchased from Macklin (Shanghai, China). Glycerol monostearate (GMS) was bought from Danisco Co., Ltd. (Shanghai, China). Corn oil (Changshouhua, Shandong, China) was obtained from a local supermarket, which was composed of 14.4% saturated fatty acids, 30.2% monounsaturated fatty acids and 55.4% polyunsaturated fatty acids. Nile red, α-amylase and mucin were obtained from Sigma-Aldrich (St. Louis, MO, USA.). Analytical grade chemicals were purchased from Beijing Chemical Works (Beijing, China). Distilled water was used throughout the study. Commercial products for lard, mayonnaise and cream cheese were purchased from the local market (Beijing, China).

### 2.2. Preparation of Oleogels and Emulsions

Oleogel preparation. EC oleogels were prepared by dissolving GMS at 80 °C and EC at 150 °C in corn oil (SCZL-2 Digital Magnetic Stirrer, Yuhua, China), and kept at 150 °C (300 rpm) for 20 min. The samples were cooled and left for 24 h to allow the development of stable gel structures. The oleogels with different contents of GMS (0, 1, 2, 5%) were termed OG0, OG1, OG2, OG5.

Emulsions preparation. To obtain the emulsions, the water phase:oil phase ratio was affixed at 3:7. The oleogel was mixed with water using an overhead stirrer (JJ-1 Precision Timing Electric Stirrer, Ronghua, China) at 300 rpm for 3 min. The emulsions with different GMS content (0, 1, 2, 5%) were termed E-OG0, E-OG1, E-OG2, E-OG5. Moreover, E-OG5 with different water content of (30, 40, 50%) were termed W30, W40, W50.

### 2.3. Characterization of Oleogels and Emulsions

#### 2.3.1. Macro Morphology and Microstructural Observation

The Macro morphology pictures of oleogels and emulsions were taken by a digital camera. Cryo-SEM (SU8010 scanning electron microscope, Hitachi, Japan) was utilized to observe the microstructures of oleogels following the method reported before [20]. Oleogels were transferred to a 4 °C refrigerator and kept for 24 h. Afterwards, isopropanol (3 mL) was applied in a drop-wise fashion over the chilled samples to remove the surface oil. Then the samples were put into a cryogenic preparation system (PP3000T, Quorum, UK), and frozen at −170 °C with liquid nitrogen. The samples were then transferred to the sample preparation unit held at −130 °C and a pressure of 10^−6^ mbar. Once fractured with a blade and coated with a layer of Au–Pd, the samples were inserted into the observation chamber equipped with an SEM cold stage module held at −125 °C. The average oil droplets were evaluated by Image J (v1.8.0, National Institutes of Health, Bethesda, Rockville, MD, USA).

Confocal laser scanning microscopy (CLSM) observation was performed to observe the microstructures of the emulsions using a Zeiss LSM 780 confocal laser scanning microscope (Carl Zeiss Microscopy Inc., Jena, Germany). The emulsions were dyed with a solution of Nile red (0.01 wt%) and set on a glass. They were observed at the excitation wavelength of 485 nm and emission wavelength of 595 nm, respectively. The diameter of water droplet was evaluated by Image J (v1.8.0, National Institutes of Health, Bethesda, Rockville, MD, USA).

#### 2.3.2. Oil Binding Capacity

Oil binding capacity (OBC) was evaluated to describe the stability of oleogels and emulsions. The samples were prepared in pre-weighted centrifuge tubes (*m_a_*). Then the centrifuge tubes (*m_b_*) were centrifuged at 10,000× *g* for 10 min. The released oil at the top of the sample was collected by a syringe, and the remaining tube was weighed (*m_c_*). OBC was calculated by the following Equation (1), respectively:

(1) $$\mathrm{OBC}=\left(1\;-\;\frac{m_{b\;}-\;m_{c}}{{(m}_{b}\;-\;m_{a})\times \mathrm{Oil}\;\mathrm{proportion}}\right)\;\times \;100\%$$

#### 2.3.3. Differential Scanning Calorimetry (DSC) Analysis

Thermal properties of the samples were evaluated using a DSC214 differential scanning calorimeter (NETZSCH, Germany). During the measurement, samples (5–12 mg) were placed in aluminum pans and sealed with aluminum lids, with an empty aluminum pan being the reference. The analysis was carried out from 30 °C to 180 °C at a heating rate of 10 °C/min.

#### 2.3.4. FTIR Spectra

FTIR spectra of EC, GMS powder, corn oil, oleogels and emulsions were measured using a Spectrum 100 Fourier Transform Infrared Spectrometer (Perkin-Elmer, UK) with a resolution of 4 cm^−1^. The samples were scanned at the wavelength range of 600~4000 cm^−1^ 64 times. FTIR spectra was then smoothed and baseline automatically corrected with the Thermo Scientific OMNIC software (v8, Thermo Fisher Scientific, Waltham, MA, USA).

#### 2.3.5. Rheological Analysis

A HAAKE IQ AIR rheometer (Thermo Scientific Inc., Karlsruhe, Germany) equipped with a steel parallel plate (40 mm diameter and 1 mm gap) was used to evaluate the viscoelastic properties of oleogels and emulsions. Each sample was equilibrated at the tested temperature for 3 min before tests. Lard, mayonnaise and cream cheese were tested as food models.

LVR. Linear viscoelastic region (LVR) of the samples was determined by strain sweep (0.01–100%) at the frequency of 1 Hz at 25 °C. Relevant parameters involved in LVR were summarized, including critical strain (γ) (a deviation of 10% from plateau value), yield stress (τ), viscoelastic modulus (G′_LVR_/G″_LVR_), damping coefficient (tanδ_LVR_ = G″/G′), and complex viscosity (η*_LVR_).

Frequency sweep. Frequency sweep was tested in the range of 0.01–100 Hz at the strain of 0.1% (within LVR) at 25 °C. The results were fitted to a power law model:

G′ = K′∙(2πf) ^n′^ (2)

where G′ was the storage modulus (Pa), f was the frequency (Hz), K′ was the power law model constants, and n′ was the frequency index.

Three-stage thixotropy. The samples were first subjected to a low-shear step (0.1 s^−1^) for 60 s, followed by a high-shear step (10 s^−1^) for 6 s, and finally a low-shear step (0.1 s^−1^) with each step performed for 60 s at 25 °C. Thixotropic recovery was calculated as the proportion of the end viscosity of the final step compared with that of the first step.

Flow behavior. Flow behavior of samples was evaluated by measuring viscosity at different shear rates (0.1 to 100 s^−1^, 37 °C).

#### 2.3.6. Textural Property

The samples were tested using a CT3 texture analyzer (Brookfield Engnieering Laboratories Inc., Middleboro, MA, USA) equipped with a cylindrical plunger (diameter: 12.7 mm). Samples and food models (lard, mayonnaise and cream cheese) were placed in 25 mL glass beakers. The tests were performed at a speed of 1 mm/s and a compression distance of 5 mm with an initial stress of 0.01 N. Hardness value was the peak force value measured during compression; adhesiveness force was the absolute value of negative area from the first compression in the force–distance curve; cohesiveness referred to the positive area ratio of the second compression to the first compression; springiness referred to the height ratio of the second compression and the first compression; chewiness was calculated from hardness × cohesiveness × springiness.

#### 2.3.7. Tribological Properties

The samples were measured using a strain-controlled rheometer (ARES G2, TA Instrument, Crawley, UK) equipped with a three-ball geometry, according to the method reported by Zhang et al. [10] with some modifications. The PDMS plate and the three-ball geometry was used to mimic the tongue surface and the palate, respectively. For better imitation of oral conditions, the PDMS plate was immersed in artificial saliva for at least 2 h [21]. The test was performed with a force of 1 N at 37 °C. Friction coefficients of the samples were recorded at an entrainment speed from 0.01 to 500 mm/s.

#### 2.3.8. Stability of Oleogels and Emulsions

The oleogels and emulsions were stored at 4 ℃. After 14-day storage, OBC of the samples was tested to describe the stability [22] (refer to the method-2.3.2. Oil binding capacity). The storage modulus at 1 Hz in the frequency sweep was evaluated (refer to the method-2.3.5. Rheological analysis), following the method reported earlier [23].

#### 2.3.9. Statistical Analysis

Statistical analysis was performed using Origin 2017. All measurements were repeated three times, unless otherwise stated. A one-way analysis of variance (ANOVA) using Duncan′s test was applied to determine significant differences between the mean values of each test. A significance level of *p* < 0.05 was applied throughout the study.

## 3. Results and Discussion

### 3.1. Properties of EC oleogels

#### 3.1.1. Morphology

**Figure 1:**
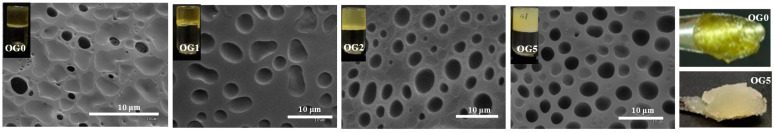
Macro morphology and Microstructures of EC oleogels with different content of GMS.

The incorporation of GMS significantly influenced the structures of the EC oleogel. All the oleogels had self-standing structures (Figure 1), due to the formation of EC networks. EC oleogel was yellowish and transparent, and the increase in GMS content resulted in the oleogels with opaque nature and milky color. Moreover, EC oleogel had coarse surface and gel fractionation, but the addition of GMS led to the formation of harder and homogeneous oleogels with smoother surface (Figure 1). Microstructural observation revealed that the oleogel was composed of polymer strands with pore size of 2~5 μm, which was in agreement with literature findings [20]. The increase in GMS content resulted in more round pores and more compact structures.

#### 3.1.2. DSC Analysis, FTIR and Oil Binding Capacity

**Figure 2:**
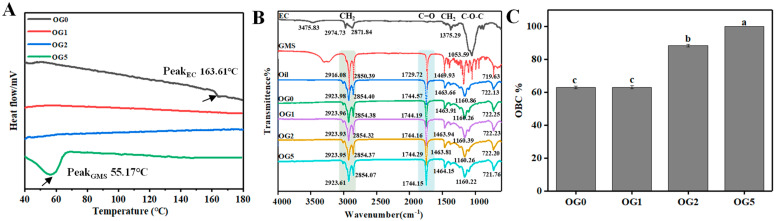
DSC (**A**), FTIR (**B**) and oil binding capacity (**C**) of EC oleogels with different content of GMS. Different lowercase letters represent significant differences (*p* < 0.05).

DSC analysis indicated that EC had a small melting temperature of ~168 °C in OG0 (Figure 2A), related to the presence of crystalline fractions, which was in agreement with literature studies [24]. The insoluble crystalline fractions of EC in the oil at 150 °C also might result in the grainy texture of OG0, as literature has reported [24]. From Figure 2A, the incorporation of GMS into the EC oleogel eliminated the crystalline fractions of EC, and thus develop more homogeneous structures. The crystallization peak (~55.17 °C) in OG5 demonstrated that excessive GMS was crystallized in the oil phase.

FTIR further revealed that there could be weakened van der Waals forces in OG1 and OG2, as the characteristic peak of CH_2_ was shifted (Figure 2B). This confirmed the interactions between GMS and the remaining hydroxyl and oxygen atoms of EC, leading to the dissolution of EC crystalline fractions [25]. CH_2_ peaks of OG2 and OG5 were shifted from 2923.96 and 2854.38 cm^−1^ to 2923.61 and 2853.07 cm^−1^, respectively. This implied the interactions between excessive GMS and triacyl-glyceride during the formation of GMS network.

Oil binding capacity was largely attributed to the interactions between liquid oil and the gelator molecules [26]. Figure 2C showed that the oleogels with higher content of GMS were capable to retain more oil. The EC oleogel without GMS had quite low OBC (~63%), suggesting poor oil–EC interactions. EC oleogel with 5% GMS was able to hold ~100% of the oil, which indicated a tighter gel network. In EC oleogels, GMS can act as a plasticizer by hydrogen-bonding with the unsubstituted hydroxyl groups on the polymer strands [25]. GMS was embedded between polymer strands, limiting the formation of junction zones between polymer strands, increasing the free volume of the system and thus modifying the EC oleogel network [22]. Second, the crystallization of GMS resulted in the development of GMS oleogels with improved oil-binding properties. Moreover, the rounder pores and more compact oleogel structures with the addition of GMS also contributed to higher oil binding capacity.

#### 3.1.3. Rheological Properties

**Figure 3:**
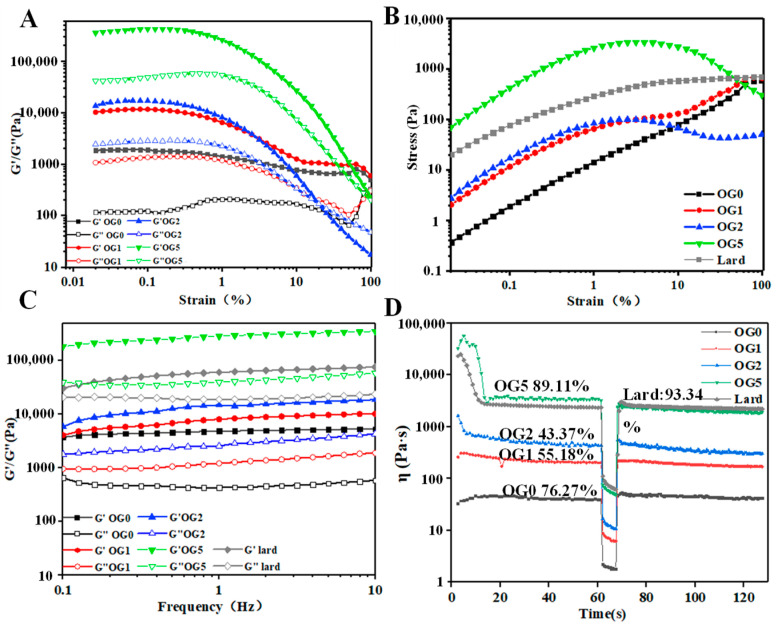
Rheological behavior of the oleogels. (**A**): Strain sweep. (**B**): Stress–strain curves obtained from strain sweeps. (**C**): Frequency sweep. (**D**): Thixotropy tests.

Rheological analysis could work to reflect the interactions and functions of structural units in colloidal systems [27]. The linear viscoelastic region (LVR) of the oleogels was lower than 1% strain (Figure 3A). Above LVR, both G′ and G′′ of oleogels rapidly declined, as the weak hydrogen bonds in EC oleogel networks were irreversibly damaged. Overall, oleogels with more GMS had higher elasticity. 1% GMS caused the increase in G′ and G′′ values by an order of magnitude, due to the hydrogen bonding between GMS and the unsubstituted OH groups of EC [28]. Only slight difference in G′ and G′′ was seen between OG1 and OG2, which could be a sign of saturated interactions among EC chains and GMS [29]. Table 1 summarized the parameters related to LVR. η*_LVR_ of oleogels was enhanced with higher GMS content, meaning improved consistency and stability [30]. The increased γ_LVR_ suggested tighter networks of the oleogels in order to resist deformation, implying stresses’ concentration within the networks [27]. Differently, OG5 had lower γ_LVR_ than that of OG0, indicating more brittle structures against deformation.

Yield stress (τ) was taken as the maximum “peak stress” beyond which flow was induced by strain, which also depicted the failure of the incipient microstructures [31]. Table 1 shows that the addition of GMS enhanced the yield stress of oleogels by over two orders of magnitude, indicating reinforced firmness against deformation. Above the yield stress, the stress was gradually decreased, indicating softening and plastic flow behaviors of the oleogels (Figure 3B). OG0 and OG1 presented straight strain–stress curves as the strain was increased, suggesting irreversible elastic deformation of EC chains. While OG2 displayed a plastic response, after reaching a maximum value, the stress displayed a plateau-like variation. However, OG5 displayed a more brittle-like response: the sample showed a more sudden drop in stress with a higher extent of deformation in post-yielding [29]. This brittle-like plastic flow during yielding gave the ductile-like behavior (spreadability) of oleogels, which was reported similarly to that of EC-lecithin oleogels [29]. The addition of GMS allowed oleogels with plastic flow characteristics, which were prone to collapse, while EC polymeric networks presented elastic deformation.

The network structures originating from hydrogen bonding of EC were responsible for the frequency dependence of the oleogels, implying weak gels (Figure 3C). As frequency was increased from 0.1 to 100 Hz, G′ was increased due to the reduction of the available relaxation time of the EC chains [30]. Moreover, GMS crystals can facilitate the formation of entanglement points in EC chains to the formation of a temporary three-dimensional network, and thus decrease the relaxation time of the EC chains. The evolution of G′ and G” with frequency fitted the Power Law model (Table 2). The increased consistency index (K′) implied higher elasticity of the oleogels with higher GMS content. n′ of oleogel was increased from OG0 to OG2, due to the plasticizing characteristic of GMS which coated the EC backbone, thus enhancing the flexibility and elasticity of EC chains. Moreover, n′ of OG5 was decreased, suggesting the weakened frequency dependence. This result was in agreement with the change in tanδ values. Tanδ values of oleogels were higher with 0–2% GMS than that with 5% GMS.

Thixotropy measurement was used to simulate the recovery percentage after instant stirring (Figure 3D). The recovery rate was higher in OG0 than those in OG1 and OG2. Although the entanglement points between GMS and EC contributed the formation of temporary knots [32], the knots were sensitive to shearing. In contrast, OG5 had much higher recovery rate. With the presence of GMS molecules, the hydrogen bonds among EC chains and GMS could be recovered when junction zones were broken after shearing [33]. Similar findings were also reported in the systems containing fat crystal-formed networks, in which the junction zones were broken down during shearing but new crystalline particles were developed by attrition [29].

The rheology properties of oleogels were compared to lard, a model plastic fat rich with saturated fats [29]. It was observed that viscoelasticity (G′, G′′, K′ and n′) of oleogel with 2–5% GMS was similar to that of the lard. EC-GMS oleogels also had similar yield stress as lard, but the yielding occurred at a lower strain. Nevertheless, the recovery rate of lard was higher (~93.34%), showing higher mechanical reversibility.

### 3.2. Properties of EC Oleogel-Based Emulsions

#### 3.2.1. Morphology

**Figure 4:**
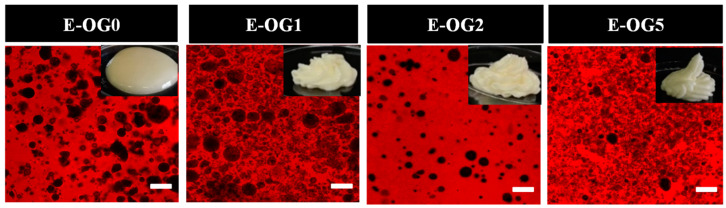
Macro-morphology and Microstructures of W/O emulsions with different content of GMS.

Figure 4 presents morphology of the emulsions containing different content of GMS. The self-supporting property of emulsions was improved with higher content of GMS, and ≥1 wt% GMS was essential for such a characteristic. CLSM observation showed the water droplets (in black) were surrounded by oil phase (in red), which confirmed the formation of W/O emulsions. The increased GMS content resulted in smaller diameter of water droplets, due to the surface activity of GMS [28].

#### 3.2.2. FTIR and Oil Binding Capacity

**Figure 5:**
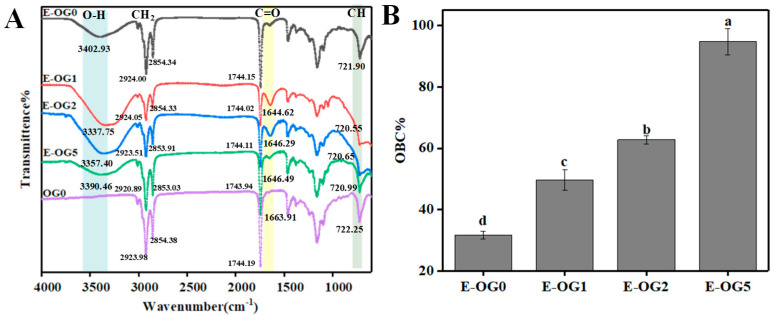
FTIR (**A**) and oil binding capacity (**B**) of W/O emulsions with different content of GMS. Different lowercase letters represent significant differences. (*p* < 0.05).

FTIR further revealed that there could be additional hydrogen bonds when water was incorporated, as the OH characteristic peak (3500–3200 cm^−1^) was vibrated (Figure 5A). Besides water–water hydrogen bonding interaction, water molecules can interact with the head groups of GMS by hydrogen bonds, thus stabilizing the emulsions [34]. In E-OG1 and E-OG2, the characteristic peak of C=O (1646 cm^−1^) stretched and the symmetric and anti-symmetric CH_2_ peaks were shifted from 2924.00 cm^−1^ and 2854.34 cm^−1^ to 2920.89 cm^−1^ and 2853.03 cm^−1^, respectively. This was a sign of van der Waals attraction related to GMS absorption onto the oil-water interface, as hydroxyl and carbonyl groups of GMS were oriented to the water side and the acyl chain was oriented to the oil phase [34]. The above findings proved that GMS was present at the interface and the continuous phase.

Moreover, higher GMS content contributed to higher oil binding capacity of emulsions (Figure 5B). E-OG0 had an OBC of 30.73 ± 1.31%, while that of E-OG5 was ~94%. This might be attributed to the collective effect of GMS both at the interface and the continuous phase.

#### 3.2.3. Rheological Properties of the Emulsions

**Figure 6:**
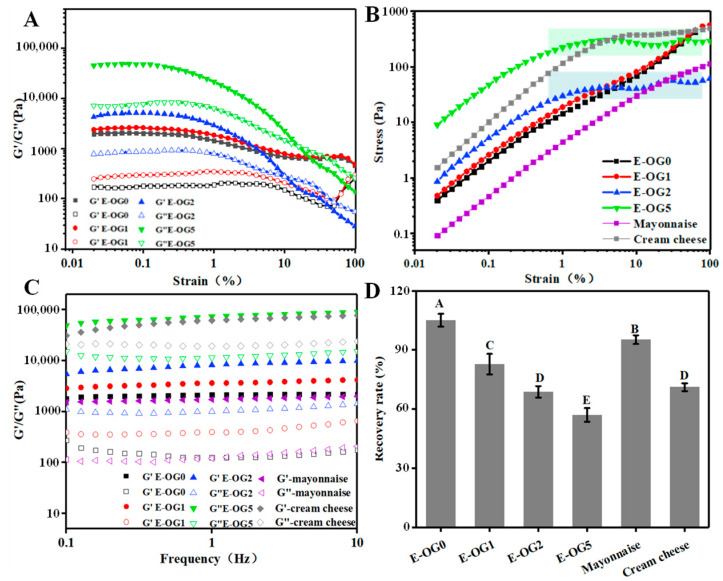
Rheological behavior of the emulsions. (**A**): Strain sweep. (**B**): Stress–strain curves obtained from strain sweeps. (**C**): Frequency sweep. (**D**): Thixotropy tests. Different uppercase letters represent significant differences. (*p* < 0.05).

The addition of GMS enhanced the viscoelasticity of the emulsions, indicated by the increased modulus (Figure 6A). Strain deformation enabled emulsions to transit from elastic-like behavior (G′ > G′′) to fluid-like (G′′ > G′) response when the strain exceeded a critical value. Lower critical stress (<1%) indicated that the structures were easy to break and then oil was released from the structures. Interestingly, E-OG0 had higher G′_LVR_, G′′_LVR_, η*_LVR_ and τ_LVR_ than OG0 (Table 1). The enhanced viscoelasticity suggested that the water droplets worked as active fillers. As for the yield stress, E-OG0 and E-OG1 presented continuous yield characteristic, similar to that of oleogels (Figure 6B). The fluidization and elastic softening of emulsions was driven by failure of the oleogel polymeric networks. In particular, E-OG2 and E-OG5 displayed two-step yielding behaviors. It was possibly due to the decomposing points of EC chains and the fluidization of the interface which allowed strain-induced breakdown [31]. This yielding behavior allowed emulsions to reach the first yield stress, and the flow initiated, while the remaining structures would enable the emulsions to hold their shapes on the point [35].

In the frequency sweep test, emulsions displayed gel-like behaviors, as Gʹ dominated Gʹʹ in the studied range (Figure 6C). It was expected that the increase in GMS concentration led to stronger structures of the emulsions. K′ values of GMS was also significantly increased (Table 1). Different from oleogels, the emulsions showed lower tanδ value as GMS content was increased (≤2%) (Table 1). All the emulsions exhibited thixotropic responses, which confirmed that the emulsions were shear-thinning. Lower recovery rate was observed in the emulsions with higher GMS content (Figure 6D). Compared to OG-0, E-OG0 had higher structural recovery (~105%), since water droplets effectively absorbed stress and hindered structural deformation. Differently, E-OG5 had a lower recovery rate (~57%). The structural breakdown of emulsions was usually attributed to the collapse of junction zones and the destruction of interface. As the junction zones originated from adjacent crystals were in a larger proportion, the junction zones were hard to recover [36].

Mayonnaise and cream cheese were evaluated as references for viscoelastic behavior and mechanical reversibility. Mayonnaise presented continuous yield behavior with an indistinct yield plateau. The viscoelasticity modulus (G′ and G′′) and K′ (~1730) of mayonnaise was similar to those of emulsions with 1~2% GMS, but mayonnaise showed higher recovery rate (~95.31%). Cream cheese revealed plastic yielding behavior, as the stress showed a plateau-like variation after the maximum value of the stress. The viscoelastic modulus, K′ value, and recovery rate (~71.17%) of cream cheese were similar to those of emulsions with 2–5% GMS. In general, the oleogel emulsions adjusted by GMS exhibited similar rheological behaviors as some commercial products.

### 3.3. Oral Processing Properties

Textural perception during oral processing was a dynamic process, which transited from mechanics behaviors (e.g., texture), rheology-dominated behaviors (e.g., shearing viscosity) to tribology-dominated behaviors (e.g., lubrication) [16]. In this part of the study, texture, viscosity and lubrication properties of the oleogels and emulsions were systematically characterized.

#### 3.3.1. Textural Properties

Textural properties of oleogels and emulsions (adhesiveness, springiness,) were enhanced when GMS content was increased (Table 3). Compared with the samples without GMS, the addition of 1% GMS reduced the hardness, but further increased in GMS content enhanced the hardness. Different from the rheology tests, the thickening effect of GMS on oleogels and emulsions was relatively small when its concentration was ≤2%. For example, the adhesiveness of OG2 was increased slightly compared to that of OG0, while the adhesiveness of OG5 was nearly 60 times higher than that of OG0. Gwartney reported that high adhesiveness was also correlated with the stickiness of the products during mastication [37]. So the higher adhesiveness of emulsions indicated that the stickiness of emulsions was higher with the increase of GMS. Springiness represented the extent of recovery after compression [38], and GMS contributed higher springiness of samples during chewing. There was no significant difference in the cohesiveness of OG0, OG1, OG2, except for OG5 (*p* < 0.05). This suggested that GMS with a content ≥2% was required to enhance the binding strength of oleogel networks, and thus strengthened their resistance against damage. In the emulsions, higher cohesiveness with increased GMS content implied stronger interactions between the structural elements. In particular, commercial products (lard, mayonnaise and cream cheese) showed significantly higher cohesiveness. Overall, lard had similar adhesiveness and hardness as OG5, but higher springiness and chewiness. Moreover, textural properties of mayonnaise were closer to E-OG2, and that of cream cheese resembled the emulsions with 2–5% GMS.

#### 3.3.2. Shearing Viscosity

**Figure 7:**
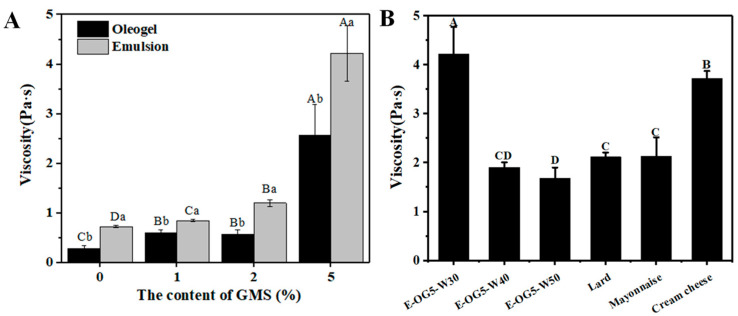
Shear viscosity as the rate of 50 s^−1^ of oleogels and emulsions (**A**) and different water content of E-OG5 and products (**B**). (**A**): Different uppercase or lowercase letters represent significant differences of GMS content or oleogels and emulsions. (**B**): Different uppercase represent significant differences of samples, respectively (*p* < 0.05).

After the first bite, food is further sheared between the surface of the tongue [39]. Viscosity tests taken at the shear rate of 50 s^−1^ highly correlated with mouthfeel [40]. In our research, higher GMS concentration led to higher viscosity of the samples. The literature has reported that viscosity related to stronger thickness perception [41]. It indicated that the increased GMS content contributed to thicker properties of the emulsions. Viscosity of the oleogels was significantly lower than that of emulsions at the same GMS content (*p* < 0.05) (Figure 7A). As water content was further increased to 50% in E-OG5 (Figure 7B), the viscosity was decreased. In spite of this, the viscosity of E-OG5 with 50% water was still higher than E-OG2. This proved the potential of the emulsions to improve the creaminess perception of low-fat food [41].

#### 3.3.3. Tribology Properties

**Figure 8:**
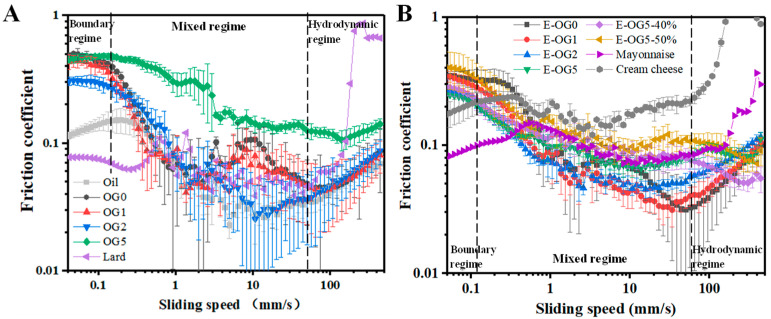
Stribeck curves were obtained for corn oil and oleogel (**A**) and emulsions (**B**).

During oral processing, the adherence of food components at the tongue surfaces was determined by the friction force, which affected sensory perception, especially smoothness [39]. Oleogels had mixed and hydrodynamic regimes, but with limited boundary regime (Figure 8A). Because oleogels could develop the entrainment film of oil, they behaved similarly to lubricated oil with no apparent boundary regime [7]. At lower sliding speed, oleogels had higher friction coefficients than corn oil, indicating thicker film in the contacting surface. As GMS content was increased, the friction coefficients of oleogels were lower suggested decreased asperity degree of the oleogels. In contrast, the addition of 5% GMS allowed a higher friction coefficient of the oleogel. As the sliding speed was increased, the oleogels were diffused to form lubrication films with released oil, resulting in a rapid decrease in friction [42]. At high sliding speed (>100 mm/s), oleogels and corn oil had similar friction coefficients, as the structures of oleogels were completely destroyed [7].

Emulsions presented similar tribology behavior to that of oleogels and oil, probably because oleogels were the continuous phase of the emulsions. Hamilton et al. also reported that the addition of W/O emulsion in gels was similar to oil, which reduced the friction coefficient of gels [43]. In the mixed regimes, the friction coefficient was also decreased at lower GMS content (≤2%), while it was increased at 5% GMS. As the water content of E-OG5 was higher (Figure 8B), the friction coefficient was increased in the boundary and hydrodynamic regime. The decreased viscosity of emulsions formed thinner films as water content was increased, thus causing water exclusion.

In the mixed regime, lard showed similar friction coefficient as oleogels with 1–2% GMS. Moreover, the friction coefficient of mayonnaise and cream cheese was close to that of E-OG5. The similar stickiness and creaminess perception of samples adjusted by GMS showed great potential as fat replacers, as GMS would form the higher surface-covered boundary film [44]. However, compared to oleogels and emulsions, commercial products (lard, mayonnaise and cream cheese) showed lower friction coefficients in the boundary regime, but higher friction coefficients in the hydrodynamic regime.

### 3.4. Stability of Oleogels and Emulsions

**Figure 9:**
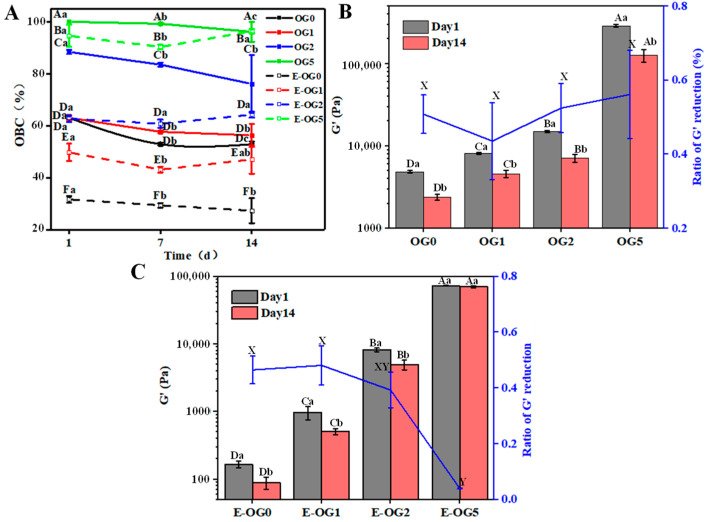
Oil binding capacity (**A**) of oleogels and emulsions. The G′ value and G′ reduction ratio of oleogels (**B**) and emulsions (**C**) was evaluated at 1 Hz of frequency sweep. Different uppercase and lowercase letters represent significant differences in different GMS content and storage time, respectively (*p* < 0.05).

The stability of oleogels and emulsions were also described by the changes in retained oil (oil binding capacity) and storage modulus during storage. As shown in Figure 9A, OBC of oleogels was enhanced as GMS was increased, due to the tighter network of the oleogels. Higher GMS content also resulted in higher oil retention, while in the EC oleogel without GMS most of the oil was released from the beginning of storage. After a 7-day storage, oleogels had higher oil loss (*p* < 0.05), which might be attributed to the weakened intermolecular interactions of EC and EC-oil interactions [22]. There was no additional oil loss of oleogels in the 14-day storage. As for the emulsions, they all had higher oil loss than the corresponding oleogels. E-OG0 had the fastest oil release, which was a sign of weak structures (~27.38% OBC after 14 days). When GMS was included, the extent of oil release was significantly reduced, as only minor oil release (~97.36% OBC after 14 days) was observed in E-OG5. Moreover, the instability (i.e., coalescence, flocculation) of oil droplets might also result in the oil loss [23].

The storage modulus at 1 Hz was tested to evaluate the strength of samples [25]. G′ of oleogel and emulsions was decreased after storage due to the oil loss (Figure 9B,C). G′ of oleogels was decreased by about 50% after a 14-day storage, since the weakened interactions between oil and EC networks resulted oil leak. Moreover, agglomeration of GMS crystals occurred during storage, resulted in a broken network with the subsequent deleterious effect in the mechanical and oil binding capacity of the oleogels [22]. In particular, G′ of E-OG5 showed insignificant change during the 14-day storage test. It was reported that EC limited the mobility of monoglyceride molecules in oleogels, as EC can slow the transition and agglomeration of crystals [22]. Moreover, the viscous and rigid continuous phase enhanced oil binding capacity, which inhibited the flow of oil droplets [42].

## 4. Conclusions

The study investigated the structural, rheological and tribological properties of EC oleogels and the corresponding W/O emulsions modulated by GMS. As GMS could interact with EC polymer network via van der Waals forces and hydrogen bond, the gel networks were enhanced, leading to higher storage modulus, viscosity and yield stress of the oleogels and emulsions. The results concluded that rheological and tribological properties of lard, mayonnaise and cream cheese could be mimicked by EC oleogels or emulsions with the addition of suitable amount of GMS, which also suggested the resembled creaminess and smoothness properties. Moreover, oleogels and W/O emulsions adjusted by GMS showed similar lubrication behavior as the oil, and W/O emulsions had favorable smoothness but with low fat content. The information obtained in this study would be beneficial to tune structure of W/O emulsions for better sensory and health attributes.

## Figures and Tables

**Table 1:** Viscoelastic parameters of the oleogels and emulsions during strain sweep.

Samples	ɣ_LVR_/%	G′_LVR_/Pa	G″_LVR_/Pa	η*_LVR_/Pa·s	Tanδ_LVR_	τ_LVR_ (Pa)
OG0	0.419 ± 0.054 ^BC^	1468.511 ± 280.721 ^D^	144.219 ± 21.496 ^D^	258.750 ± 10.960 ^D^	0.103 ± 0.009 ^D^	5.958 ± 1.384 ^D^
OG1	0.627 ± 0.009 ^A^	7699.102 ± 367.695 ^C^	1174.193 ± 183.141 ^C^	1196.333 ± 123.747 ^C^	0.154 ± 0.010 ^C^	49.675 ± 1.944 ^C^
OG2	0.622 ± 0.015 ^A^	10,275.510 ± 359.917 ^B^	2400.212 ± 219.203 ^B^	1606.103 ± 166.170 ^B^	0.227 ± 0.015 ^A^	64.345 ± 6.003 ^B^
OG5	0.455 ± 0.017 ^B^	197,250.890 ± 230,163.257 ^A^	54,400.002 ± 3818.377 ^A^	56,001.610 ± 1751.411^A^	0.163 ± 0.004 ^B^	1529.503 ± 112.429 ^A^
E-OG0	0.429 ± 0.021 ^b^	1838.552 ± 210.011 ^d^	179.150 ± 7.282 ^d^	268.601 ± 24.890 ^d^	0.100 ± 0.003 ^d^	6.883 ± 1.332 ^d^
E-OG1	0.406 ± 0.019 ^b^	2181.213 ± 118.087 ^c^	300.453 ± 19.021 ^c^	338.504 ± 36.062 ^c^	0.126 ± 0.012 ^c^	8.638 ± 0.479 ^c^
E-OG2	0.623 ± 0.014 ^a^	3596.131 ± 9.192 ^b^	827.502 ± 51.619 ^b^	564.850 ± 35.143 ^b^	0.231 ± 0.008 ^a^	22.230 ± 1.739 ^b^
E-OG5	0.273 ± 0.082 ^c^	40,906.303 ± 1829.992 ^a^	8096.003 ± 244.659 ^a^	6479.333 ± 508.410 ^a^	0.189 ± 0.007 ^b^	89.520 ± 3.564 ^a^

Different uppercase and lowercase letters represent significant differences of oleogels and emulsions, respectively. (*n* = 3, *p* < 0.05).

**Table 2:** The Power Law parameters (K’ and n′) of the oleogels and emulsions.

Samples	K′	n′	R^2^
OG0	4743 ± 40.820 ^E^	0.067 ± 0.002 ^E^	0.966
OG1	7553 ± 45.393 ^D^	0.166 ± 0.002 ^B^	0.969
OG2	13,330 ± 50.866 ^C^	0.176 ± 0.007 ^A^	0.954
OG5	276000 ± 220.276 ^A^	0.124 ± 0.012 ^CD^	0.983
lard	57,340 ± 516.210 ^B^	0.148 ± 0.010 ^C^	0.960
E-OG0	159.7 ± 37.70 ^f^	0.220 ± 0.005 ^a^	0.994
E-OG1	886.2 ± 59.74 ^e^	0.217 ± 0.008 ^a^	0.994
E-OG2	7927 ± 52.01 ^c^	0.112 ± 0.009 ^c^	0.984
E-OG5	71,009 ± 160.97 ^a^	0.115 ± 0.007 ^c^	0.987
Mayonnaise	1730 ± 60.21 ^d^	0.057 ± 0.002 ^d^	0.999
Cream cheese	8660 ± 68.17 ^b^	0.165 ± 0.011 ^b^	0.997

The equation was fit for G′ = K′(2πf)^n′^. Different uppercase or lowercase letters represent significant differences of oleogels and lard or emulsions and mayonnaise and cream cheese, respectively. (*n* = 3, *p* < 0.05).

**Table 3:** The textural properties of the oleogels and emulsions.

Sample	Hardness (N)	Adhesiveness (mJ)	Springiness (mJ)	Cohesiveness	Chewiness (N)
OG0	0.045 ± 0.005 ^D^	0.055 ± 0.005 ^C^	0.034 ± 0.005 ^D^	0.26 ± 0.05 ^C^	0.045 ± 0.007 ^D^
OG1	0.033 ± 0.0043 ^E^	0.070 ± 0.011 ^C^	0.056 ± 0.006 ^C^	0.21 ± 0.04 ^C^	0.023 ± 0.009 ^E^
OG2	0.066 ± 0.0090 ^C^	0.12 ± 0.0082 ^B^	0.079 ± 0.008 ^C^	0.27 ± 0.025 ^C^	0.067 ± 0.005 ^C^
OG5	2.00 ± 0.17 ^A^	2.98 ± 0.22 ^A^	1.14 ± 0.19 ^B^	0.43 ± 0.08 ^B^	1.62 ± 1.01 ^B^
Lard	1.51 ± 0.071 ^B^	2.76 ± 0.15 ^A^	1.80 ± 0.51 ^A^	0.68 ± 0.069 ^A^	6.04 ± 0.90 ^A^
E-OG0	0.15 ± 0.030 ^c^	0.11 ± 0.034 ^f^	0.037 ± 0.074 ^e^	0.027 ± 0.004 ^e^	0.014 ± 0.004 ^e^
E-OG1	0.09 ± 0.0042 ^d^	0.17 ± 0.042 ^e^	0.087 ± 0.010 ^d^	0.043 ± 0.0094 ^d^	0.32 ± 0.029 ^d^
E-OG2	0.14 ± 0.0094 ^c^	0.34 ± 0.017 ^d^	0.24 ± 0.025 ^c^	0.090 ± 0.0082 ^c^	0.46 ± 0.056 ^c^
E-OG5	0.54 ± 0.052 ^a^	0.76 ± 0.06 ^a^	0.61 ± 0.027 ^a^	0.31 ± 0.026 ^b^	2.56 ± 0.21 ^a^
Mayonnaise	0.14 ± 0.021 ^c^	0.43 ± 0.04 ^b^	0.21 ± 0.02 ^c^	0.63 ± 0.05 ^a^	0.47 ± 0.092 ^c^
Cream cheese	0.37 ± 0.031 ^b^	0.51 ± 0.059 ^b^	0.22 ± 0.041 ^b^	0.65 ± 0.038 ^a^	1.10 ± 0.14 ^b^

Different uppercase and lowercase letters represent significant differences of oleogels and emulsions, respectively. (*n* = 5, *p* < 0.05)^.^

## Data Availability

Data is contained within the article.
